# A Beneficial Plant-Associated Fungus Shifts the Balance toward Plant Growth over Resistance, Increasing Cucumber Tolerance to Root Herbivory

**DOI:** 10.3390/plants11030282

**Published:** 2022-01-21

**Authors:** Loren J. Rivera-Vega, John M. Grunseich, Natalie M. Aguirre, Cesar U. Valencia, Gregory A. Sword, Anjel M. Helms

**Affiliations:** 1 Department of Biological Sciences, Virginia Polytechnic Institute and State University, Blacksburg, VA 24060, USA; lorenrv@vt.edu; 2 Department of Entomology, Texas A&M University, College Station, TX 77843, USA; johngrunseich@tamu.edu (J.M.G.); entomip2000@tamu.edu (C.U.V.); gasword@tamu.edu (G.A.S.); 3 Ecology and Evolutionary Biology Program, Texas A&M University, College Station, TX 77843, USA; n.aguirre@tamu.edu

**Keywords:** *Acalymma vitattum*, *Cucumis sativus*, growth-defense tradeoff, growth-differentiation balance, induced systemic resistance, plant-growth promoting fungus

## Abstract

Plants allocate their limited resources toward different physiological processes, dynamically adjusting their resource allocation in response to environmental changes. How beneficial plant-associated microbes influence this allocation is a topic that continues to interest plant biologists. In this study, we examined the effect of a beneficial fungus, *Phialemonium inflatum,* on investment in growth and anti-herbivore resistance traits in cucumber plants (*Cucumis sativus*). We inoculated cucumber seeds with *P. inflatum* spores and measured several growth parameters, including germination rate, above and belowground biomass, and number of flowers. We also examined plant resistance to adult and larval striped cucumber beetles (*Acalymma vitattum*), and quantified levels of defense hormones in leaves and roots. Our results indicate that *P. inflatum* strongly enhances cucumber plant growth and reproductive potential. Although fungus treatment did not improve plant resistance to cucumber beetles, inoculated plants were more tolerant to root herbivory, experiencing less biomass reduction. Together, these findings document how a beneficial plant-associated fungus shifts plant investment in growth over herbivore resistance, highlighting the importance of microbes in mediating plant-herbivore interactions. These findings also have important implications for agricultural systems, where beneficial microbes are often introduced or managed to promote plant growth or enhance resistance.

## 1. Introduction

Plants dynamically allocate or distribute their resources across multiple physiological processes—including growth, reproduction, and responses to biotic and abiotic stresses. Determining the factors that influence resource allocation patterns is a topic that has long interested plant ecologists. Some of these factors include resource availability (e.g., nutrients and water) [1], exposure to stress (biotic and abiotic) [2,3], ontogeny [4,5,6], genotype [7,8], domestication [9], and presence or absence of microbial relationships [10]. The growth-differentiation hypothesis aims to predict how plants invest resources between growth-related and differentiation-related processes in different environmental conditions [11,12]. Growth refers to processes involving cell division and elongation (e.g., production of roots or leaves), while differentiation refers to processes that enhance the structure and function of different cells (e.g., trichome production or increased cuticle thickness). Differentiation also encompasses plant investment in chemical defense traits, such as production of enzymes, phytohormones, and chemical compounds involved in defense [13]. Tradeoffs between growth and differentiation can occur due to limited resources, conflict in molecular or metabolic pathways involved in these processes, or when traits involved are genetically linked [14,15]. Here, we examine how a beneficial plant-associated fungus affects plant investment in growth and defense against herbivory.

Beneficial plant-associated microbes, including many species of bacteria and fungi, are known to have widespread effects on plant growth and defense. These changes can occur separately or simultaneously and through different mechanisms. For example, some species of plant growth-promoting bacteria (PGPB) increase plant growth by facilitating resource acquisition [16] or modulating plant hormone levels tied to growth processes [17]. Bacteria in the genus *Azospirillum* are known to fix nitrogen in the soil, thus increasing the availability of this important resource to plants [18]. On the other hand, some species like *Pseudomonas putida,* produce the plant growth hormone indole-3-acetic acid (IAA), which directly stimulates root growth, and additionally increases the plant’s ability to absorb nutrients [19]. Beneficial microbes can also affect plant defenses through multiple mechanisms, including producing defense metabolites [20] and triggering induced systemic resistance (ISR) [21]. ISR is an enhanced plant defense capacity against a broad range of biotic stressors induced by beneficial microbes [21]. Beneficial microbes in plant roots can induce or prime higher levels of plant hormones so that when plants are challenged by an herbivore or pathogen, they can mount a faster and stronger defense response [22,23]. For example, beneficial *Trichoderma* fungi trigger ISR by modulating expression of genes involved in jasmonic acid, salicylic acid, and ethylene signaling, increasing plant resistance to attack by pathogens and herbivores [24,25,26]. Although numerous studies have now documented beneficial effects of plant-associated microorganisms, the specific roles these microbes play in regulating tradeoffs between plant growth and defense remain unclear. Furthermore, context dependency is likely to occur through interactions among resource availability, enhanced growth processes, production of defenses, and environmental stressors.

Recently, our lab isolated a strain of the fungus *Phialemonium inflatum* that improves both growth and anti-herbivore resistance in cotton plants, seemingly overcoming tradeoffs between these processes. Inoculation with *P. inflatum* confers enhanced plant resistance against a variety of herbivores, including root knot nematodes [27,28], cotton bollworms [29], cotton aphids [30], and Lygus bugs [31]. Notably, this fungus has also been shown to increase cotton plant growth [29]. An outstanding question is whether similar beneficial fungus-mediated effects occur in other agriculturally important plant species. *P. inflatum* is a well-known fungal endophyte and epiphyte which has been isolated from a variety of unrelated plant species [32,33]. The goal of this study was to evaluate how fungal inoculation affects growth-defense tradeoffs in cucumber plants (*Cucumis sativus*) by determining the influence of *P. inflatum* on (1) plant growth and reproduction, and (2) plant anti-herbivore resistance. We hypothesized that the growth vs. defense dynamics would be affected by the presence of *P. inflatum*. Based on our previous work with cotton, we predicted that fungus-inoculated cucumber plants would have both enhanced growth and increased resistance against striped cucumber beetles (*Acalymma vittatum*). To test these predictions, we quantified cucumber growth traits and resistance against cucumber beetle adults and larvae. As biochemical indicators of herbivore-induced defenses, we also measured levels of the defense signaling molecules jasmonic acid (JA) and salicylic acid (SA) in the absence and presence of beetle herbivory. Although JA and SA are typically associated with plant defense, they can also be involved in signaling related to plant growth and association with beneficial microbes, and previous studies have reported microbial effects on induced plant defenses and their associated signaling pathways [34,35]. This research documents evidence for microbe-enhanced plant growth and herbivory tolerance and provides further insights into the role of beneficial microbes in modulating tradeoffs in plant investment in growth and defense processes.

## 2. Results

### 2.1. Phialemonium Inflatum Increases Cucumber Plant Germination, Biomass, and Reproduction Potential

**Figure 1:**
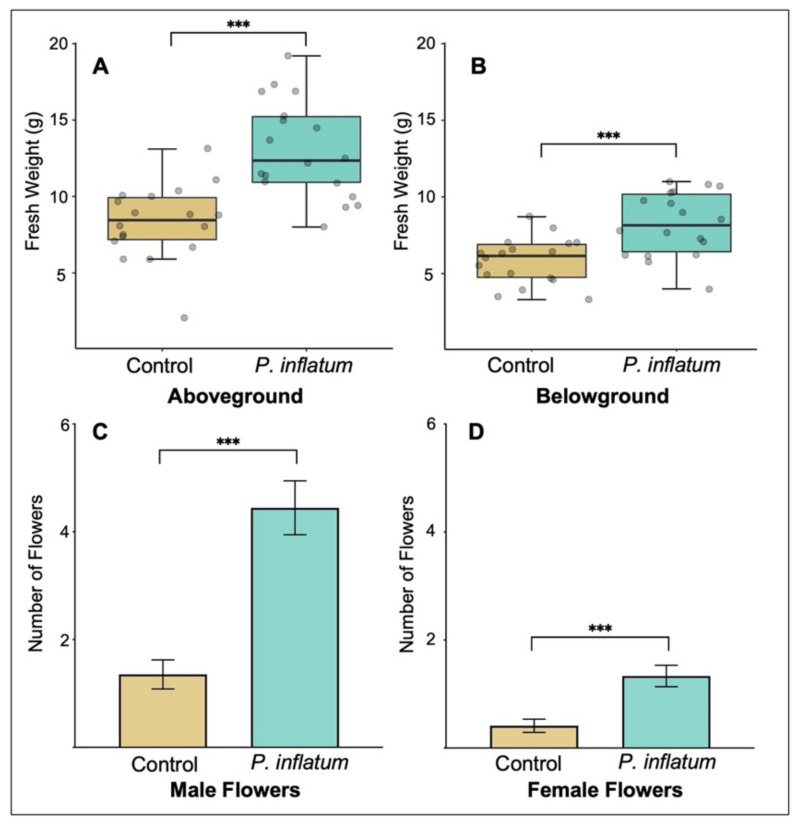
(**A**) Aboveground and (**B**) belowground biomasses were higher in plants grown from seeds inoculated with *Phialemonium inflatum* spores. The boxes represent the interquartile range that contains values between the 25th and 75th percentile. The inside line denotes the median. The numbers of (**C**) male and (**D**) female flowers were higher in plants inoculated with *P. inflatum*. N = 18 for all treatments. Means ± SE are presented. (*** *p* < 0.001).

Cucumber plants inoculated with *Phialemonium inflatum* had a higher germination rate compared to untreated control plants. We observed 100% germination for seeds treated with *P. inflatum* and only 60% germination in control seeds. Treatment with *P. inflatum* also increased cucumber plant biomass. Both aboveground biomass (Student’s *t*-test, *T* = −5.02, *p* < 0.001, Figure 1A) and belowground biomass (Student’s *t*-test, *T* = −4.00, *p* < 0.001, Figure 1B) were higher in *P. inflatum*-treated plants compared to untreated controls. Furthermore, plants inoculated with *P. inflatum* produced significantly more flowers compared to untreated control plants, suggesting potential for higher reproduction and future yield. This was true for both male (GLM, *T_1,36_* = 5.11, *p* < 0.001, Figure 1C) and female (GLM, *T_1,36_* = 15.20, *p* = 0.001, Figure 1D) flowers.

### 2.2. Seed Treatment with Phialemonium Inflatum Reduces Cucumber Plant Resistance to Adult Cucumber Beetles

**Figure 2:**
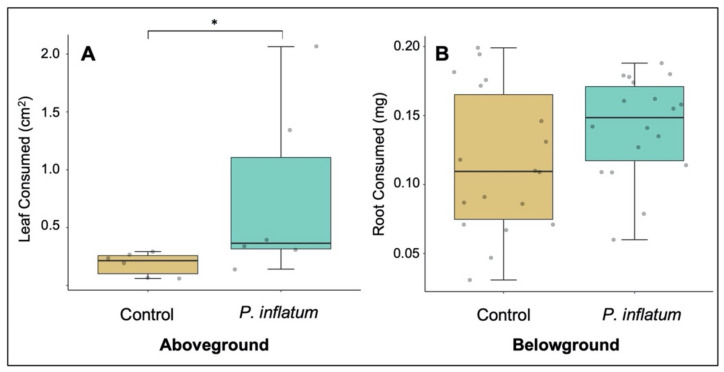
(**A**) Leaf area consumed by adult beetles was higher on fungus-treated plants. (**B**) Root mass consumed by beetle larvae was similar on *P. inflatum*-treated and control plants. N = 6 for aboveground treatments and N = 18 for belowground treatments. The boxes represent the interquartile range that contains values between the 25th and 75th percentile. The line inside the box denotes the median. The error bars show the largest/smallest observation that is less than or equal to the upper quartile plus/minus 1.5 the length of the interquartile range. (* *p* < 0.05).

In a no-choice experiment, adult cucumber beetles consumed significantly more leaf tissue on plants treated with *P. inflatum* compared to control plants (Wilcoxon-Mann-Whitney W = 4, *p* = 0.026, Figure 2A). Furthermore, in a two-choice experiment, more adult beetles preferred fungus-treated over control plants (χ^2^ = 31.14, *p* < 0.001) with 56% settling on treated plants, 33% on controls, and 11% not choosing either treatment. In contrast, cucumber beetle larvae consumed similar amounts of root tissue (Student’s *t*-test, *T* = −1.72, *p* = 0.095, Figure 2B) on treatment and control plants. We also measured larval performance and found no difference in mass gain while feeding on fungus-treated or control plants (Student’s *t*-test, *T* = 0.32, *p* = 0.74).

### 2.3. Cucumber Plants Treated with Phialemonium Inflatum Were more Tolerant to Root Herbivory

**Figure 3:**
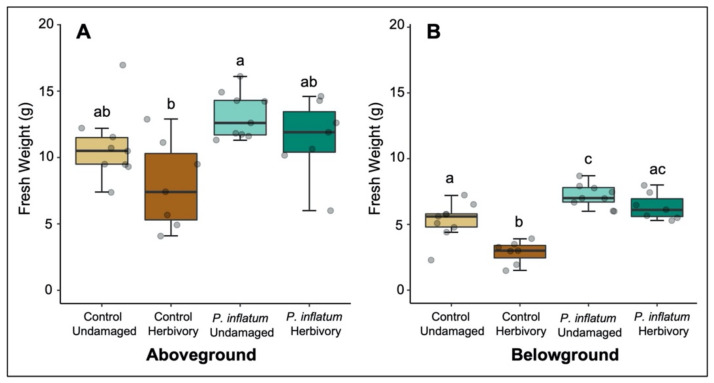
(**A**) Above and (**B**) belowground biomasses from control and inoculated plants in the presence and absence of belowground herbivory. Control plants experienced more tissue loss from herbivory than plants inoculated with *Phialemonium inflatum.* The boxes represent the interquartile range that contains values between the 25th and 75th percentile. The line inside the box denotes the median. The error bars show the largest/smallest observation that is less than or equal to the upper quartile plus/minus 1.5 the length of the interquartile range. N = 9 for all treatments. Different letters indicate statistically significant differences, *p* < 0.05.

When plants were challenged with cucumber beetle larvae belowground, *P. inflatum*-treated plants showed higher tolerance to herbivory by exhibiting improved growth relative to untreated control plants. There was a trend toward improved tolerance in aboveground tissues of fungus-treated plants (Student’s *t*-test (Control vs. Control + Herbivory), *T* = 1.88, *p* = 0.08; Student’s t-test (*P. inflatum* vs. *P. inflatum* + Herbivory), *T* = 1.36, *p* = 0.20, Figure 3A). Root biomass of fungus-treated plants was higher compared to control plants (Student’s *t*-test (Control vs. *P. inflatum*), *T* = −3.10, *p* = 0.007, Figure 3B). Notably, root herbivory significantly reduced the root biomass of control, but not *P. inflatum*-treated plants (Student’s *t*-test (Control vs. Control + Herbivory), *T* = 4.22, *p* = 0.001; Student’s *t*-test (*P. inflatum* vs. *P. inflatum* + Herbivory) *T* = 1.69, *p* = 0.11, Figure 3B), indicating that fungal treatment helped the plants tolerate root damage. Herbivory reduced root biomass by 26% in untreated plants, but only by 12% in plants inoculated with *P. inflatum*.

### 2.4. Roots but Not Leaves of Cucumber Plants Treated with Phialemonium Inflatum had Compromised Defense Responses to Herbivory

**Figure 4:**
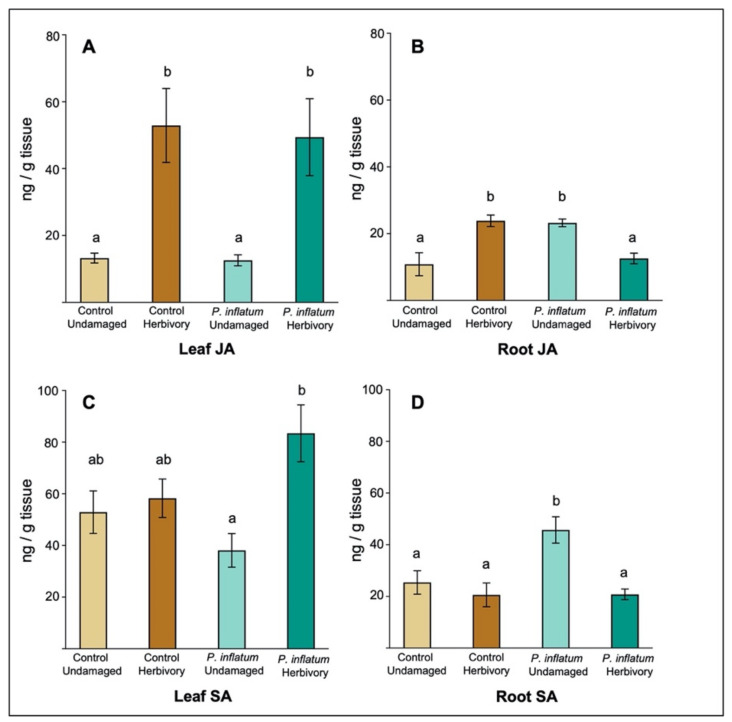
*Phialemonium inflatum* seed treatment had differing effects on plant defense signaling with and without herbivory. (**A**) Aboveground levels of JA increased with herbivory but were not affected by fungal treatment. (**B**) Belowground levels of JA were higher in *P. inflatum*-treated plants and reduced with herbivory. (**C**) Aboveground levels of SA increased in *P. inflatum*-treated plants following herbivory. (**D**) Belowground levels of SA increased with *P. inflatum* treatment and were suppressed following herbivory. Data shown are untransformed, but statistical analyses were performed on log transformed data. N = 17 for all treatments. Means ± SE are presented. Different letters indicate significant differences, *p* < 0.05.

To determine whether *P. inflatum* seed treatment affects the induction of defense-related phytohormones in cucumber, we quantified levels of jasmonic acid (JA) and salicylic acid (SA) in leaf and root tissues, with and without cucumber beetle herbivory. SA is associated with plant defense against biotrophic plant pathogens and is commonly induced in plant tissues following pathogen infection, signaling the activation of downstream anti-pathogen defense responses. JA is associated with plant defense against chewing herbivores and is typically induced in response to herbivore wounding, which activates downstream anti-herbivore defenses [36,37]. The induction dynamics of these phytohormones have been well characterized across many plant species with various pathogen or herbivore attackers, as well as for plants associated with beneficial microbes [21,25]. We predicted that *P. inflatum* would increase JA induction and associated defenses in treated cucumber plants making them more resistant to cucumber beetle herbivory. However, contrary to our predictions, *P. inflatum* treatment had no effect on either the constitutive or herbivore-induced levels of JA in cucumber leaves relative to control plants without fungus. Constitutive JA levels were similar in fungus-treated and control leaves, and adult beetle herbivory induced similarly high levels of JA in both treatments (Kruskal-Wallace test, chi-sq = 25.90, *p* < 0.001, Figure 4A). Constitutive levels of SA in leaves were also similar for *P. inflatum*-treated and control plants, however, adult cucumber beetle herbivory induced higher levels of SA in *P. inflatum*-treated plants (ANOVA *F_3,65_* = 5.66, *p* = 0.001, Figure 4C), possibly suggesting these plants had elevated pathogen-associated defenses. In plant roots, constitutive JA levels were higher in fungus-treated plants compared to controls but were suppressed following herbivory by beetle larvae (ANOVA *F_3,25_* = 8.98, *p* < 0.001, Figure 4B). Constitutive root SA levels were also higher in *P. inflatum*-treated plants, and lower following herbivory by cucumber beetle larvae (ANOVA *F_3,25_* = 6.19, *p* < 0.001, Figure 4D), suggesting these plants had impaired defenses against both herbivores and pathogens belowground.

### 2.5. Phialemonium Inflatum Grows on the Surface of Cucumber Roots, but Does Not Colonize Cucumber Tissues

**Figure 5:**
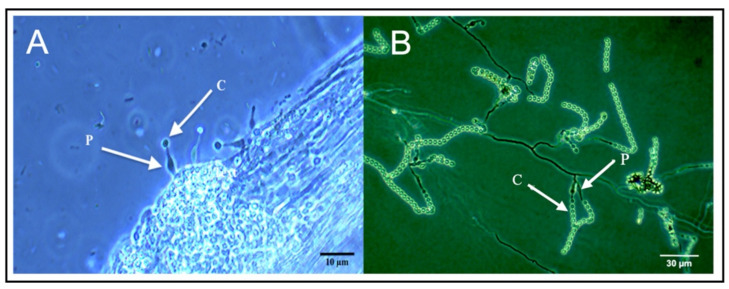
*Phialemonium inflatum* phialide (P) and conidia (C) growing on the surface of cucumber seedling roots (**A**) and on a slide culture (**B**) as seen on a phase contrast microscope.

*Phialemonium inflatum* grows on the surface of cucumber roots, but not within root, shoot, or leaf tissues. Following plating of surface-sterilized tissues from inoculated plants, *P. inflatum* was not recovered from any leaf, stem, or root samples. Microscopy revealed that hyphae and reproductive structures of *P. inflatum* grew on the surface of roots (Figure 5). However, we observed no evidence of endophytic growth.

## 3. Discussion

Plants must allocate limited pools of resources towards a variety of processes, including growth, defense, and reproduction, resulting in potential tradeoffs among these processes [38]. There is clear evidence that plants can adjust their allocation patterns dynamically in response to changes in their environments. Many past studies have focused on plant investment in growth or defense processes in response to individual stressors, such as pathogen infection or herbivory [15,39], or have considered how plant microbial associations affect specific processes [40] and there is rapidly growing interest in bringing these research fields together. Here we expand on previous work by investigating how plant interactions with a beneficial fungus affect plant resource allocation patterns toward growth and defense. Our findings demonstrate that microbes, like *P. inflatum,* can modulate plant investment in different processes, affecting plant interactions with other organisms (e.g., herbivores) and plant fitness potential. Furthermore, our results indicate that beneficial microbes can have variable effects on different plant species, and possibly different genotypes or cultivars within species, necessitating careful consideration in plant-microbe pairings to achieve predictable effects in different environments. Overall, our results highlight the complexity of plant-microbe interactions and the need for continued research in this area.

Based on our previous research with *P. inflatum* in cotton, we predicted that this fungus would promote growth, while also increasing resistance against herbivory in another plant species, cucumber [29]. As predicted, we found that *P. inflatum* increased cucumber plant growth (Figure 1). However, contrary to our predictions, fungus-treated cucumber plants were less resistant to adult cucumber beetle herbivory than untreated control plants (Figure 2). This finding was somewhat surprising as previous studies documented enhanced resistance of *P. inflatum*-treated cotton plants to a variety of different herbivore species [28,29,30,31]. Furthermore, previous research with other microbes reported that cucumber plants treated with PGPR suffered less damage from cucumber beetles [41,42]. These findings highlight species-specific responses of cucumber plants to both microbial inoculants and specialist cucumber beetle herbivores. In line with these results, a meta-analysis reported that plants associated with mycorrhizae commonly have negative effects on generalist herbivores and positive effects (e.g., higher growth rate, consumption, fecundity, etc.) on specialists [43]. Striped cucumber beetles (*Acalymma vittatum*) are specialist herbivores of plants in the family Cucurbitaceae, and our previous research suggests indirect defenses (i.e., recruitment of herbivore-killing natural enemies with volatile compounds) may be more effective against cucumber beetles than other induced defenses (e.g., toxic metabolites) [44,45]. In this study, it appears that *P. inflatum*-induced changes to cucumber plant chemistry and/or nutrition positively affected the performance of specialist cucumber beetles, whereas in previous research with cotton, *P. inflatum*-associated plants were more resistant against generalist herbivores. Notably, *P. inflatum*-inoculated cucumber plants were also more attractive to adult cucumber beetles, suggesting that fungal treatment alters plant traits associated with herbivore foraging (e.g., visual, olfactory, and gustatory cues), which has been observed for a variety of other plant-associated microbes [46]. We hypothesize that the observed differences in plant resistance are related to the type of association between *P. inflatum* and each plant species. *P. inflatum* colonizes cotton tissues as a facultative endophyte, and can be present within leaf, stem, and root tissues [28,30]. Here, we found no evidence for endophytic growth for *P. inflatum* in cucumber and only observed fungal growth on the surface of roots (Figure 5). It is possible that because endophytes colonize the plant, and are more intimately associated with plant tissues, they are more likely to induce resistance against biotic stress [47] compared to an epiphyte. Taken together, these patterns of variation clearly illustrate the important role of context-dependency in determining the ecological outcome of specific plant-herbivore-microbe interactions [48].

Although *P. inflatum*-inoculation did not enhance cucumber plant resistance against striped cucumber beetle herbivory, and increased plant susceptibility to adult beetles, we observed that fungus-treated plants were more tolerant to cucumber beetle root herbivory. This suggests that the fungus mediated a shift in defensive strategy in treated cucumber plants to enhance tolerance. Tolerance is the degree to which a plant can regrow after herbivory to reduce the effects of herbivory on plant fitness [49]. This can include directing plant resources to compensatory growth (vegetative or reproductive) [50] and/or moving nutrients away from attacked tissues [51]. In our system, cucumber plants, which already had enhanced growth by *P. inflatum*, responded to cucumber beetle root herbivory by further increasing growth, rather than switching to induction of defensive compounds and resistance. A recent study also found that increased growth led to higher herbivory tolerance—wild parsnips grow larger in their invasive range, which confers greater tolerance to specialist webworms [52]. Tolerance has been suggested to play an important role in plant defense against specialist herbivores, as specialists are often less susceptible to plant resistance traits [53]. Tolerance strategies are also predicted to be more common than induced resistance against root-feeding herbivores, many of which are specialists and have more limited mobility to emigrate compared to foliar feeding species [54]. This is likely also the case for specialist striped cucumber beetles, which are adapted to tolerate cucurbit chemical defenses [40,41]. Another recent study also reported enhanced tolerance to root herbivory in microbe-associated plants. In rice, association with the root fungal endophyte, *Piriformospora indica*, made plants more tolerant to herbivory by the belowground specialist rice water weevil (*Lissorhoptrus oryzophilus*) [55]. Overall, our results support the hypothesis that compensatory growth and tolerance are important defense mechanisms against specialist root herbivores [56] and that beneficial microbes may enhance these strategies.

We observed a reduction in resistance to adult beetle herbivory in *P. inflatum*-treated plants, however, the specific mechanisms underlying these results remain unclear. Fungal inoculation did not affect levels of the defense signaling molecules JA or SA in foliar tissues (Figure 4). However, as previously noted, chemical defenses may be less effective in conferring resistance against the specialist beetles. We suggest that future studies should examine changes in plant nutrient content following *P. inflatum* inoculation, as nutrients could play a role in mediating these interactions and the growth-promoting fungus could aid in plant nutrient uptake. In contrast to leaves, we observed evidence of altered defense signaling in cucumber roots. We measured higher constitutive levels of the phytohormones JA & SA in *P. inflatum*-treated plants. However, upon belowground herbivory by cucumber beetle larvae, these compounds were suppressed to levels like untreated control roots (Figure 4). Although JA and SA are frequently associated with plant defense against herbivores [45] and pathogens [57], they can also be involved in signaling related to plant growth processes [34,58,59]. For example, in sunflowers, inhibition of JA increased lateral and primary root growth [60], whereas in tobacco, increased levels of JA inhibited root growth [55,56]. We suggest this shift in phytohormone levels in fungal-treated plants could facilitate increased plant growth as a mechanism of tolerating root herbivory by a specialist herbivore. Future research could build on these findings by characterizing the dynamics of these and other defense or growth-related phytohormones from initial fungal colonization through initial and sustained herbivore feeding.

Possible mechanisms underlying *P. inflatum*-induced plant growth include promotion of nutrient uptake [61], modulation of plant signaling metabolites, and microbial production of growth-promoting compounds. Notably, *P. inflatum* was observed growing ectophytically as an epiphyte (Figure 5), peripherally around cucumber roots after seed treatment and not endophytically as was observed in cotton [28,29,30]. This suggests *P. inflatum* could enhance cucumber growth by increasing the absorptive surface area around roots, allowing for more efficient transfer of nutrients, water, and other resources to the plant [62,63]. Additionally, some fungal species can alter plant growth by synthesizing growth-promoting compounds that are recognized by the root system. In fact, multiple fungi produce growth promoting hormones such as gibberellins and auxins [64]. Indeed, there is a growing appreciation for the complex and non-mutually exclusive ways that fungal seed treatments can mediate plant phenotypic responses in the absence of direct colonization [65]. In the case of *P. inflatum*, further research is clearly needed to tease apart the specific mechanisms of plant growth promotion when growing as an epiphytic versus endophytic plant-associated fungus in different plant species.

The use of beneficial microbes to improve sustainable practices in agriculture has increased significantly in the last decade. The current research contributes to a better understanding of basic plant physiological mechanisms in plant-microbe-herbivore interactions and can also help identify potential new tools for crop improvement. We observed that *P. inflatum* increases cucumber growth and flower counts, which are important traits for improving crop yields. We also found that fungus-treated plants were more tolerant to root herbivory, which may also be an important component of pest management against specialist root-feeding insects. Beneficial soil-borne microbes can promote plant growth and reproduction, increase nutrient use efficiency, and protect against pathogens and pests, suggesting great potential for harnessing these effects to benefit agricultural production systems.

## 4. Materials and Methods

### 4.1. Plants, Fungus, and Insects

Cucumber plants (*Cucumis sativus* cv. Max Pack) were grown from non-sterilized seeds and used in experiments after reaching the second true-leaf stage (Johnny’s Selected Seeds, Fairfield, ME, USA). Plants were grown in individual pots in topsoil mix (Hyponex Corporation, Marysville, WA, USA) with 3 g Osmocote^®^ fertilizer (15-9-12 N-P-K) (Scotts, Marysville, WA, USA) and were kept in an insect-free, climate-controlled growth room with lighting conditions of 16 h light: 8 h dark, at approximately 22 °C and 56% RH (Fluence, Austin, TX, USA). Striped cucumber beetles (*Acalymma vittatum*) were maintained in a laboratory colony on cultivated squash (*Cucurbita pepo* cv. Raven) and cucumber. The *Phialemonium inflatum* (TAMU490) strain used was originally isolated from cultivated cotton in the field [27] and grown for these experiments in 100 × 15 mm plastic Petri dishes (VWR International, Radnor, PA, USA) containing potato dextrose agar (Hardy Diagnostics, Santa Maria, CA, USA) in the dark at 28 °C for three weeks. Mature cultures were used to make spore suspensions. For spore collection, 20 cc of a 0.01% *v/v* Triton X-100 (Millipore Sigma, MA, USA) solution was added to the Petri dishes containing the fungus, then a sterile L-shaped scraper was used to remove conidia from the surface. This concentrated spore suspension was filtered using 200 and 500 mesh (75 and 25 µm respectively) 76 mm diameter sieves (Gilson Inc., Lewis Center, OH, USA). A Neubauer hemocytometer and a bright field microscope (400× magnification) were used to calculate spore concentration, then the corresponding amount of 0.01% *v/v* Triton X-100 solution was added to adjust final spore concentration to 1 × 10^7^ cells/mL. Seeds of cucumber plants were treated with 1 mL of spore solution or 1 mL of deionized autoclaved water at the time of sowing.

### 4.2. Plant Growth and Reproductive Potential

To determine the influence of *P. inflatum* seed treatment on cucumber growth and potential reproduction, we quantified plant germination rates, biomass, and numbers of male and female flowers for fungus-treated and control plants. Cucumber seeds were sown in topsoil mix and treated with *P. inflatum* as described above. The percent germination was recorded for fungus treated (*n* = 18) and control plants (*n* = 18). To assess plant growth effects, plants were grown as previously described until they reached the three-leaf stage. Then they were harvested, roots were washed, and root and shoot biomasses were recorded (fresh weight). In a separate experiment, to assess fitness effects, cucumber plants were grown until flowers appeared, approximately 4 weeks after sowing. We counted the numbers of male and female flowers present for fungus-treated (*n* = 18) and control (*n* = 18) plants.

### 4.3. Adult Cucumber Beetle Feeding and Preference

To determine the influence of *P. inflatum* seed treatment on cucumber plant resistance to adult cucumber beetles, we quantified beetle feeding damage in a no-choice experiment and preference in a two-choice experiment. Fungus-treated and control plants were grown as previously described until they reached the three-leaf stage. In the no-choice test, we caged five adult beetles on each plant (*n* = 6) using a mesh bag (10 cm × 10 cm) and allowed them to feed for 24 h. Beetles were starved for 24 h prior to the experiment. After 24-h of feeding, we removed the beetles and harvested the leaves and calculated the area consumed using ImageJ software (National Institutes of Health, Bethesda, USA). In the two-choice test, we placed five adult beetles (starved for 24 h) in mesh cages (30 cm × 30 cm × 30 cm), each with one control and one fungus-treated plant (*n* = 9). Beetles were allowed to settle on plants and feed for 24 h then preference was recorded as % beetles on each treatment.

### 4.4. Larval Cucumber Beetle Feeding and Performance

To determine if *P. inflatum* seed treatment affects cucumber plant resistance to cucumber beetle larvae, we quantified the amount of root tissue larvae consumed and the percent mass gain of larvae on fungus-treated (*n* = 18) and control (*n* = 18) plants. Cucumber plants were grown as described above until the four-leaf stage. Plant roots were harvested, washed, and cut-root assays were conducted. Fresh root cuttings were weighed and placed in sterile Petri dishes on 1% agar. Individual second-instar cucumber beetle larvae were weighed and introduced to each plate. Plates were sealed with parafilm, and larvae were allowed to feed for 48 h. Remaining root mass and larval mass gain were then recorded. To account for possible mass loss due to desiccation, roots from agar plates with no larvae were also weighed after 48 h. 

### 4.5. Plant Tolerance to Herbivory by Cucumber Beetle Larvae

To determine if cucumber plants inoculated with *P. inflatum* were more tolerant to herbivory by cucumber beetle larvae, we measured plant biomass for fungus-treated and control plants with and without larval herbivory (*n* = 9). Plants were grown as described above until the four-leaf stage, then half of the fungus-treated and control plants were each challenged with five second-instar cucumber beetle larvae, while the other half remained undamaged. After larvae fed for 7 days, we harvested the plants, washed the roots, and recorded their mass (fresh weight).

### 4.6. Plant Defense Signaling (Phytohormones)

To determine the influence of *P. inflatum* seed treatment on cucumber defenses, we quantified levels of the defense hormones jasmonic acid (JA) and salicylic acid (SA) with or without cucumber beetle herbivory. All samples were collected at 12:00 to avoid differences in phytohormone levels due to circadian rhythms and from leaves of the same age to avoid differences due to ontogeny. Aboveground leaf and belowground root tissues were collected and processed separately as follows.

Leaf tissue: Plants were grown as described above until the four-leaf stage, then half of the fungus-treated and control plants were each challenged with adult cucumber beetles, while the other half remained undamaged (*n* = 17). For foliar herbivory treatments, adult beetles were starved for 24 h, then a single beetle was caged on one leaf per plant using a mesh bag (10 cm × 10 cm) and were allowed to feed for 24 h [66,67]. Control plants were caged with empty bags. We collected a leaf tissue sample from each plant (~100 mg), which was flash-frozen in liquid nitrogen and stored at −80 °C until analysis.

Root tissue: As above, half of the fungus-treated and control plants were challenged by cucumber beetle larvae, while the other half remained undamaged (*n* = 17). For root herbivory treatments, five second-instar beetle larvae were added to the roots of each plant and allowed to feed for 24 h. After 24 h, roots were harvested and washed. We collected a root tissue sample from each plant (~100 mg), which was flash-frozen in liquid nitrogen and stored at −80 °C until analysis.

The plant hormones jasmonic acid (JA) and salicylic acid (SA) were measured as indicators of plant defensive status and the strength of defense induction in response to herbivore feeding damage [37]. Extraction and quantification of JA and SA was conducted as previously described in Schmelz et al. [36,68]. Plant hormones were extracted and derivatized to methyl esters, which were then isolated using vapor-phase extraction. The compounds were analyzed by GC/CI-MS (Agilent Technologies, Santa Clara, CA, USA) using isobutane and selected ion monitoring (SIM). We quantified amounts of jasmonic acid by adding 100 ng dihydro-JA to each sample as an internal standard and salicylic acid by adding 100 ng 2-Hydroxybenzoic Acid-d6 to each sample as an internal standard. The presence of these compounds was confirmed by comparing the retention times and spectra of the samples with standards of the compounds.

### 4.7. Phialemonium Inflatum Growth and Colonization of Cucumber Tissues

Seeds of cucumber were planted in 6.5 cm^2^ pots containing 250 cc of a mixture of sterilized 80% sand and 20% peat moss (*n* = 10). One mL of a *P. inflatum* spore suspension containing 1 × 10^7^ conidia/mL was added on top of seeds, then seeds were covered with soil mixture. Controls were treated with 1 mL of a 0.01 TritonX-100 solution. Plants were kept for two weeks in a room at 30 °C, 60% RH and 14:10 light and dark photoperiod. At the end of the two-week period, seedlings (first true leaf fully expanded) were uprooted then the shoot was separated from the root and placed in individual Ziploc bags. Surface sterilization was done following Zhou et al. [69]. Imprints of leaves and roots were taken from each sample to validate the effectiveness of surface sterilization. Leaves were cut into 9 pieces of approximately 0.8 cm^2^ and placed on PDA media along with two 1 cm pieces of stem. Roots were cut into 9 pieces of approximately 1 cm and plated on a different PDA dish. Petri dishes were sealed with parafilm and kept in the dark for seven days. We inoculated extra PDA dishes with spores of *P. inflatum* as a reference for fungal colony size and development to compare with putative *P. inflatum* colonies isolated from plant tissues.

### 4.8. Microscopic Observations of P. inflatum on Cucumber Roots

Surface sterilized seeds were grown on sterile solid media containing Murashige and Skoog (MS) basal salt (3 g/L) (Sigma–Aldrich, St. Louis, MO, USA) and agar (10 g/L) contained in sterile mason jars for 9 days. Seedlings were removed from MS media and dipped in a sterile plastic jar containing a 1 × 10^6^ *P. inflatum* spore suspension that was previously incubated for 24 h at 26 °C in the dark. Roots were soaked for 10 min and then transferred to a new plastic cup, sealed, and kept at room temperature for 3 days. Pieces of 1 cm root were placed on a slide and observed at 400× magnification with a phase contrast microscope.

### 4.9. Statistical Analyses

Statistical analyses were performed using the software program R (R Version 3.6.1, R Core Team, 2019). Residuals were checked for normality. To meet the assumptions of normality, data were either log transformed, or a non-parametric test was used. For biomass assays and individual tolerance comparisons, we used Student’s t-tests to determine statistical differences. Generalized linear models with a Poisson distribution were used to compare flower counts. A Mann Whitney test was used to compare adult feeding assays. One-way ANOVAs and Tukey post hoc tests were used to analyze above- and belowground biomass, tolerance, and phytohormone levels. Leaf JA was analyzed using Kruskal-Wallis one-way analysis of variance followed by a Dunn’s Test. Differences were considered statistically different at *p*-values < 0.05.

## Data Availability

The data presented in this study are available on request.
